# Author Correction: gp130/STAT3 signaling is required for homeostatic proliferation and anabolism in postnatal growth plate and articular chondrocytes

**DOI:** 10.1038/s42003-022-03167-5

**Published:** 2022-02-25

**Authors:** Nancy Q. Liu, Yucheng Lin, Liangliang Li, Jinxiu Lu, Dawei Geng, Jiankang Zhang, Tea Jashashvili, Zorica Buser, Jenny Magallanes, Jade Tassey, Ruzanna Shkhyan, Arijita Sarkar, Noah Lopez, Siyoung Lee, Youngjoo Lee, Liming Wang, Frank A. Petrigliano, Ben Van Handel, Karen Lyons, Denis Evseenko

**Affiliations:** 1 Department of Orthopaedic Surgery, Keck School of Medicine of USC, University of Southern California (USC), Los Angeles, CA 90033 USA; 2 Department of Orthopaedic Surgery, Nanjing First Hospital, Nanjing Medical University, Nanjing, 210006 Jiangsu China; 3 Department of Orthopaedic Surgery, Zhongda Hospital, School of Medicine, Southeast University, Nanjing, 210009 Jiangsu China; 4 Department of Orthopedics, The Affiliated Jiangning Hospital with Nanjing Medical University, Nanjing, 211100 Jiangsu China; 5 Department of Orthopaedic Surgery, Sir Run Run Hospital, Nanjing Medical University, Nanjing, 211166 Jiangsu China; 6 State Key Laboratory of Oral Diseases, Department of Oral and Maxillofacial Surgery, West China Hospital of Stomatology, Sichuan University, 610041 Chengdu, China; 7 Department of Radiology, Keck School of Medicine of USC, University of Southern California (USC), Los Angeles, CA 90033 USA; 8 Department of Orthopaedic Surgery, David Geffen School of Medicine, University of California Los Angles (UCLA), Los Angeles, CA USA; 9 Institute of Digital Medicine, Nanjing Medical University, Nanjing, 210006 Jiangsu China; 10 Department of Stem Cell Research and Regenerative Medicine, USC, Los Angeles, CA 90033 USA

**Keywords:** Stem-cell differentiation, Cartilage development, Bone development

Correction to: *Communications Biology* 10.1038/s42003-021-02944-y, published online 17 January 2022.

The original version of this Article contained an error in Fig. 1f, in which the labels for pSTAT3, STAT3 and Histone H3 were omitted. The correct version of Fig. 1f is:
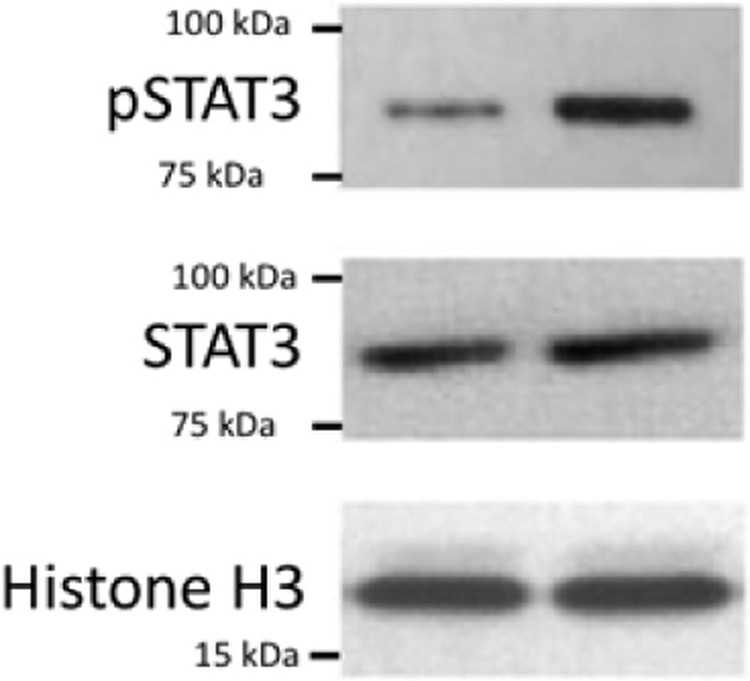


which replaces the previous incorrect version:



The error/errors have been corrected in both the PDF and HTML versions of the Article.

