# Supplementary material for: gp130/STAT3 signaling is required for homeostatic proliferation and anabolism in postnatal growth plate and articular chondrocytes
Source: Commun Biol. 2022 Jan 17;5:64. doi: 10.1038/s42003-021-02944-y (PMC8763901; doi:10.1038/s42003-021-02944-y)
Supplement: Supplementary file 1 — Supplementary Information [file 42003_2021_2944_MOESM1_ESM.pdf]

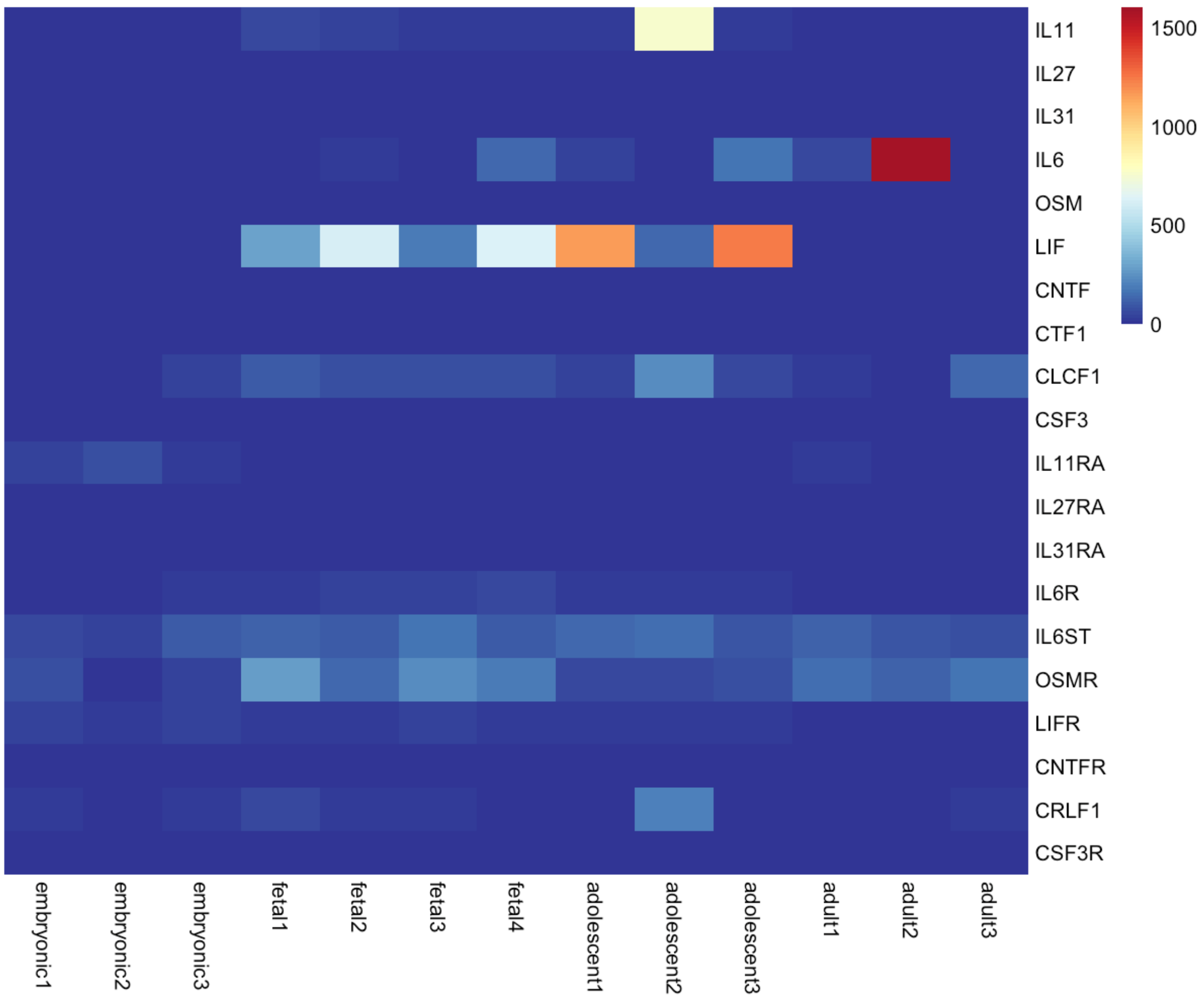

Supplementary Figure 1: Expression patten of IL-6 family cytokines and receptors during human ontogeny. Analysis of RNA-sequencing data from 4 stages of human ontogeny[12] demonstrated that gp130 (IL6ST) is expressed at all stages of development, while expression of LIF is enriched at fetal and adolescent stages when growth plates are active in humans. Normalized expression values are shown.

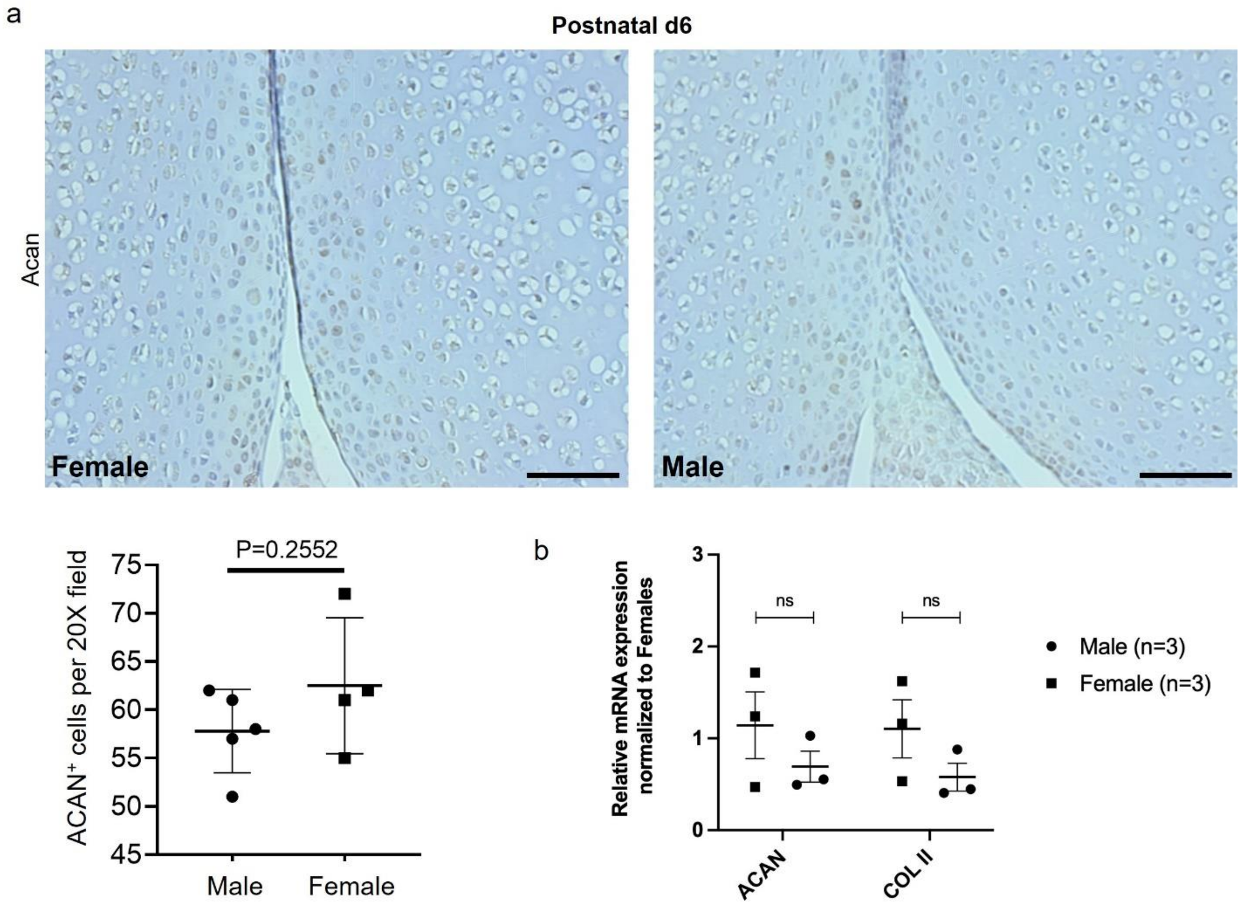

Supplementary Figure 2: Levels of aggrecan protein and mRNA are not different between females and males at the time of Cre induction. (a) Immunohistochemistry of knee joints for Acan at postnatal day 6 (P6) demonstrated no significant differences between wild type females and males. n = 4-5. (b) qPCR for *Acan* and *Col2a1* on chondrocytes isolated from wild type mouse femoral heads showed similar expression levels between females and males. n = 3; scale bars = 50 µm.

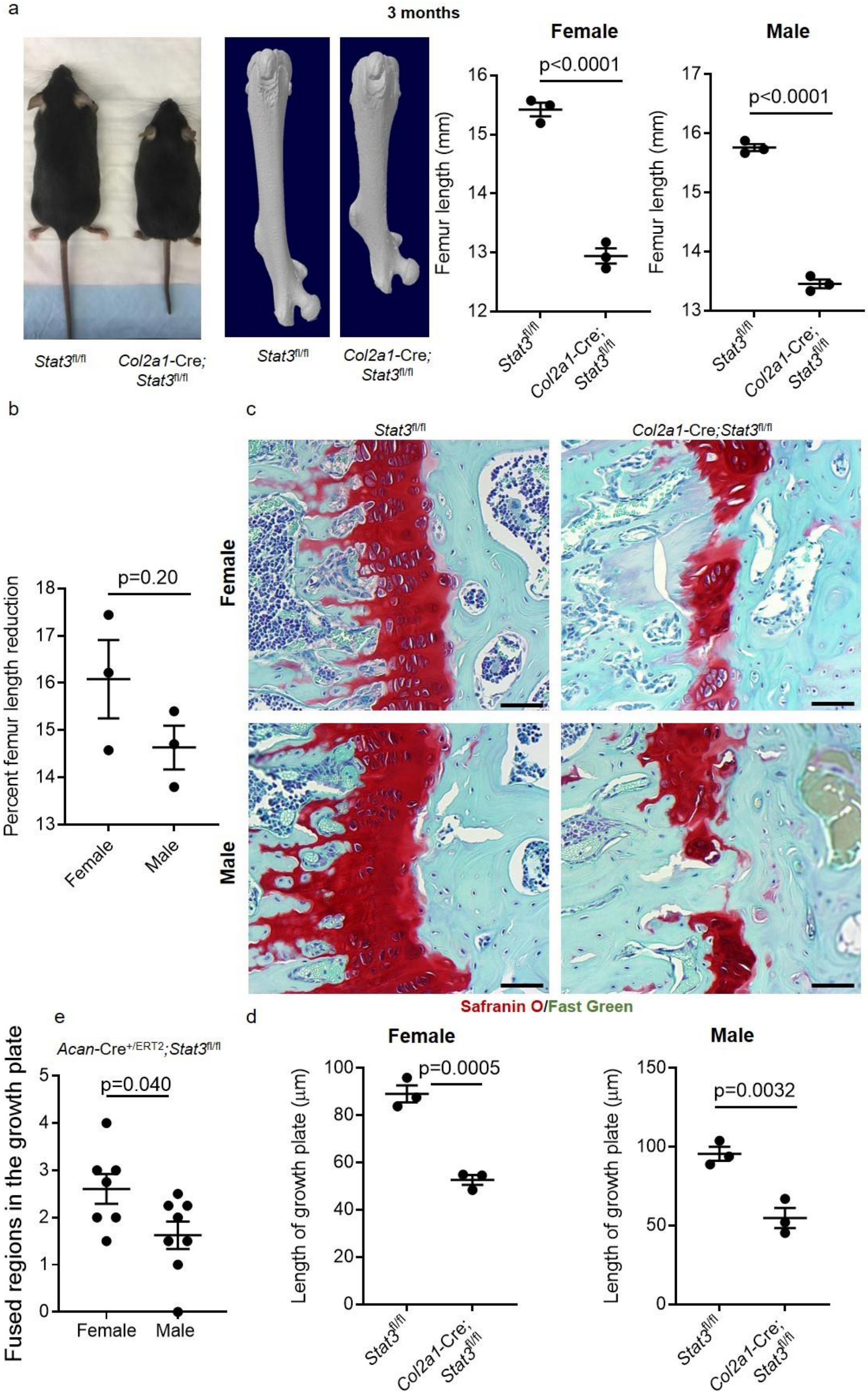
a
3 months
Female
Male
p<0.0001
p<0.0001
Femur length (mm)
Stat3fl/fl
Col2a1-Cre; Stat3fl/fl
b
p=0.20
Percent femur length reduction
Female
Male
c
Stat3fl/fl
Col2a1-Cre;Stat3fl/fl
Female
Male
Safranin O/Fast Green
e
Acan-Cre+/ERT2;Stat3fl/fl
p=0.040
Fused regions in the growth plate
Female
Male
d
Female
Male
p=0.0005
p=0.0032
Length of growth plate (μm)

Supplementary Figure 3: Constitutive deletion of *Stat3* in chondrocytes via *Col2a1*-Cre resulted in reduced body size and growth plate fusions at 3 months. (a) Analysis of control *Stat3*$^{fl/fl}$ and constitutive *Col2a1*-Cre;*Stat3*$^{fl/fl}$ female and male mice at 3 months demonstrated substantially smaller body size and statistically significant reductions in femoral length (b) as determined by microCT. (c) Histological assessment (Safranin O/Fast green staining) of growth plates revealed reduced thickness (d) and fusions in both female and male mice. For all experiments, n = 3; scale bars = 50 µm. (e) *Acan*-Cre$^{+/ERT2}$;*Stat3*$^{fl/fl}$ females evidenced significantly more frequent fusions of the growth plate at 3 months versus *Acan*-Cre$^{+/ERT2}$;*Stat3*$^{fl/fl}$ males; n = 7-8.

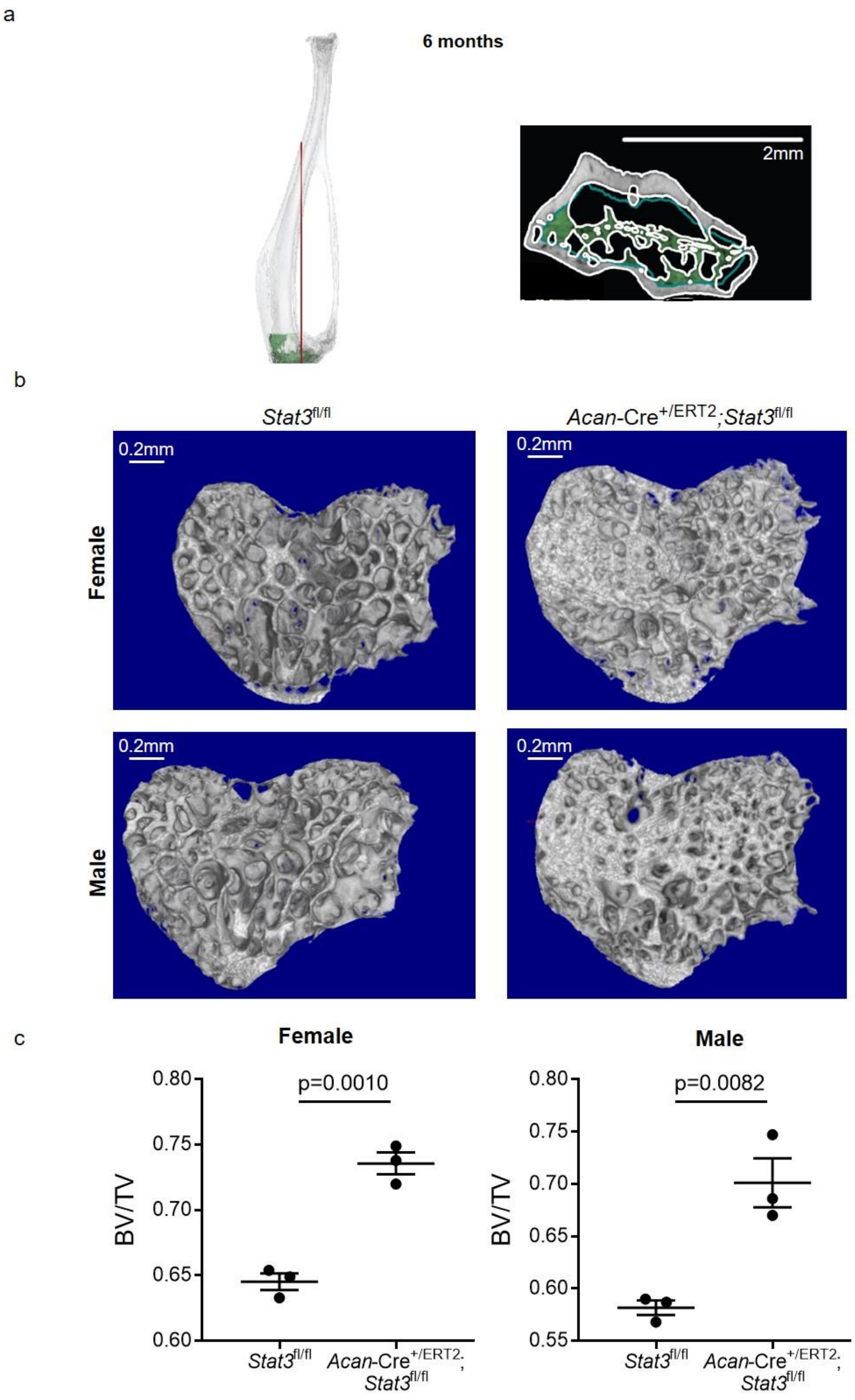

a
6 months
2mm
b
Stat3fl/fl
Acan-Cre+/ERT2;Stat3fl/fl
Female
Male
0.2mm
c
Female
Male
p=0.0010
p=0.0082
BV/TV
0.80
0.75
0.70
0.65
0.60
0.55
Stat3fl/fl
Acan-Cre+/ERT2;
Stat3fl/fl

Supplementary Figure 4: Early postnatal deletion of *Stat3* in chondrocytes increased bone density at 6 months. (a) microCT analysis of control *Stat3*$^{fl/fl}$ and *Acan*-Cre$^{+/ERT2}$;*Stat3*$^{fl/fl}$ mice induced with tamoxifen at P2/P3 revealed loss of *Stat3* increased bone density in the proximal tibial epiphyseal region. The red line on the 3D tibia surface rendering demonstrates the location of transversal cut to visualize bounders between cortical and trabecular bone for region of interest (ROI) segmentation. The 2D green shaded area represents a section of the 3D ROI used to calculate bone volume to total volume (BV/TV). (b) Representative 3D renderings of the ROI in the proximal tibial epiphysis used to calculate BV/TV (c) in for both female and male mice of the indicated genotypes. For all experiments, n = 3.

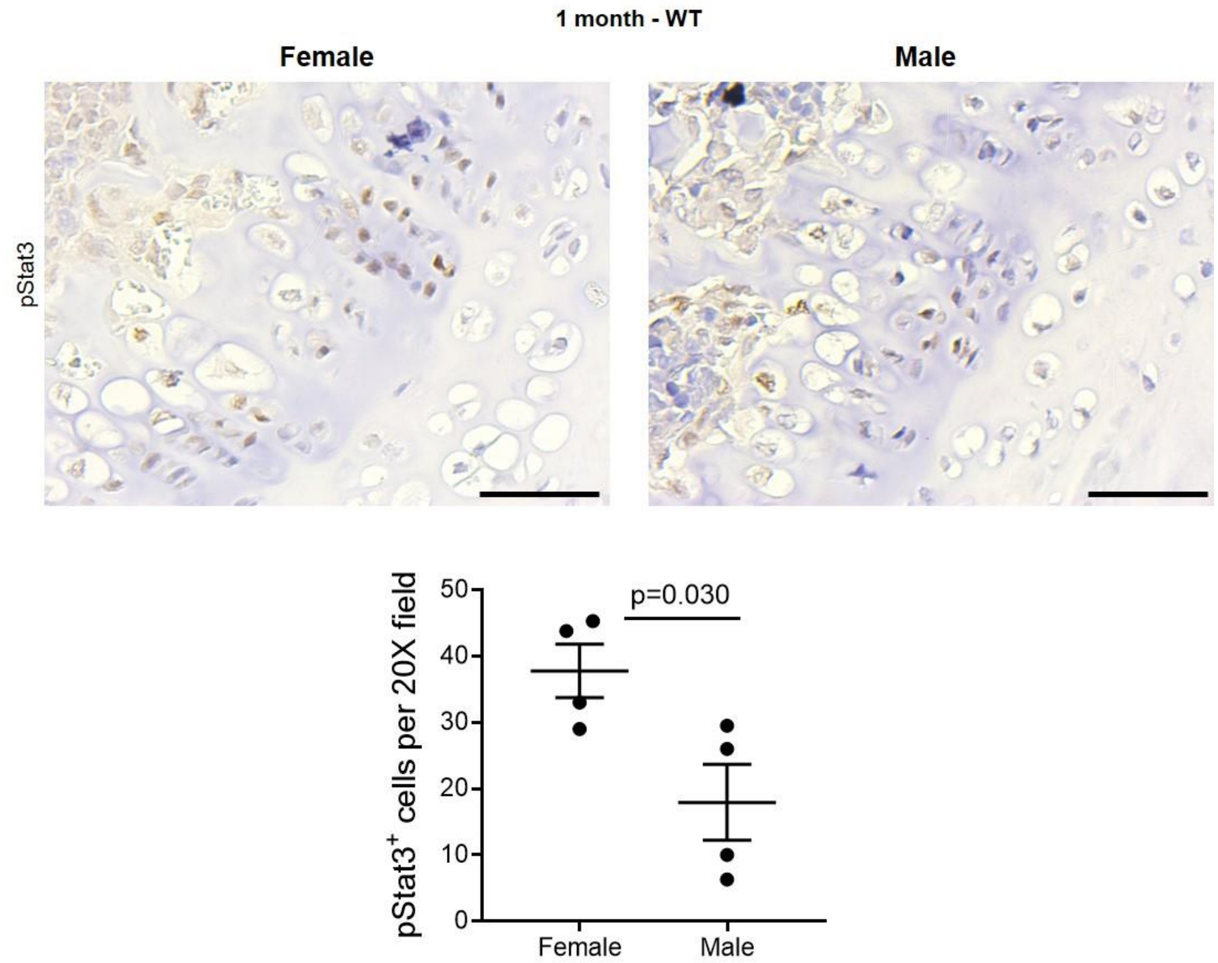

Supplementary Figure 5: Stat3 activity is enriched in the vertebral growth plates of female versus male wild type mice. Immunohistochemistry for activated Stat3 (pStat3) in vertebral growth plates of wild type female and male mice at 1 month of age demonstrated significantly more active Stat3 in female mice. Note the localization of pStat3 in the proliferative zone. n = 4; scale bars = 50 µm.

	Name	Descriptions	Abbreviation
1	Ventral (anterior) vertebral body height[1]	Vertebral body height, measured from ventral view	VVBH
2	Dorsal (posterior) vertebral body height[1]	Vertebral body height, measured from dorsal view	DVBH
3	Cranial transversal diameter of the vertebral body[1]	Transverse (mediolateral) diameter of the vertebral body. Measured on the cranial view from the external cortex of the right border to the external cortex of the left border.	CTD
4	Cranial DV(AP) diameter of the vertebral body[1]	Dorsoventral (anteroposterior) diameter of the vertebral body. Length measured on the cranial at the midline of the vertebral body from the external cortex of the anterior (ventral) border to the external cortex of the posterior (dorsal) border.	CDVD
5	Caudal transversal diameter of the vertebral body[1]	Transverse (mediolateral) diameter of the vertebral body. Measured on the caudal view from the external cortex of the right border to the external cortex of the left border.	CaTD
6	Caudal DV(AP) diameter of the vertebral body[1]	Dorsoventral (anteroposterior) diameter of the vertebral body. Length measured on the caudal at the midline of the vertebral body from the external cortex of the anterior (ventral) border to the external cortex of the posterior (dorsal) border.	CaDVD
7	Cranial endplate surface area[2]		CSA
8	Caudal endplate surface area[2]		CaSA

[1]measurement in mm; [2]measurement in mm^2

Supplementary Table 1: Measurement description on the lumbar vertebrae (L1-L6). Landmark-based data were collected from the lumbosacral region and pelvic girdle. Eight measurements were collected for each lumbar vertebra and then used to generate the data in Supplementary Data 2.

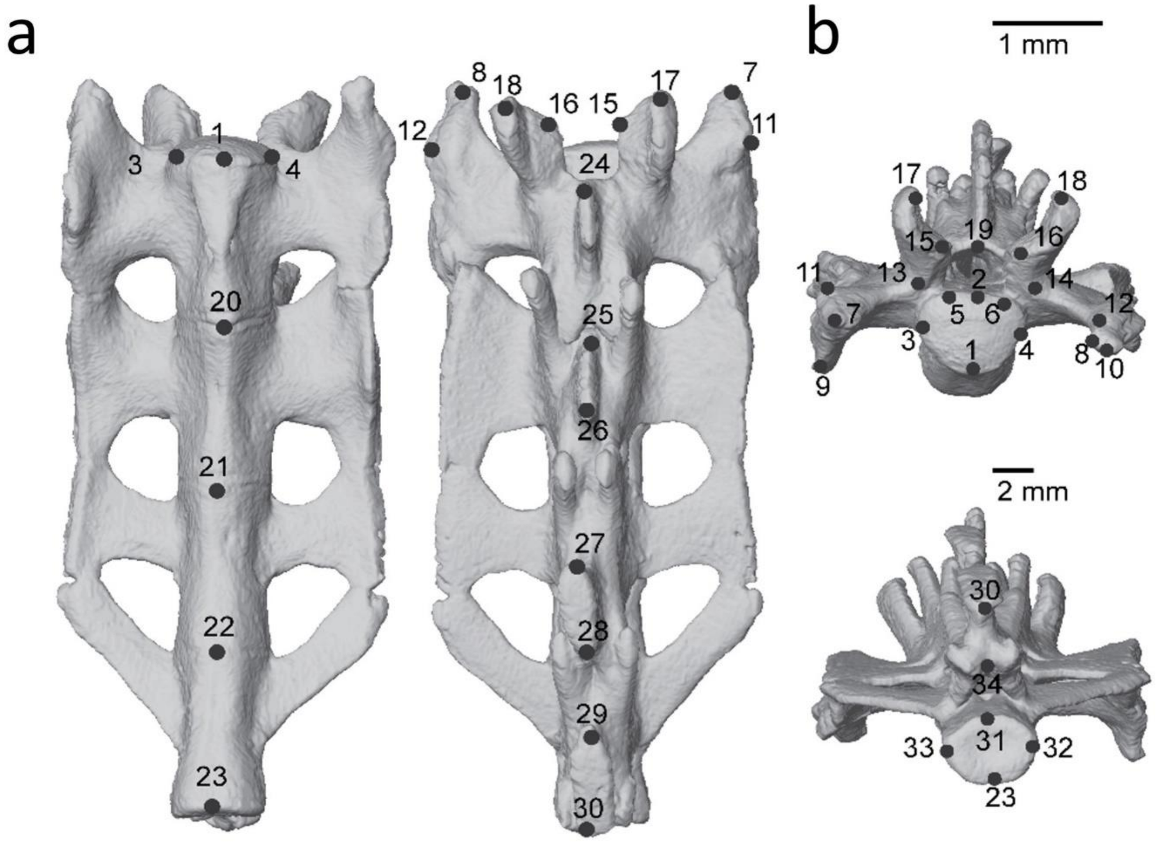

Supplementary Figure 6: Sacrum of a male *Acan*-Cre$^{+/ERT2}$;*Stat3*$^{fl/fl}$ mouse illustrating the 33 landmarks used as a basis for geometric morphometrics. (a) Ventral and dorsal views. (b) Cranial and caudal views. Landmarks are described in the Materials and methods section. Imaging was conducted at 6 months.

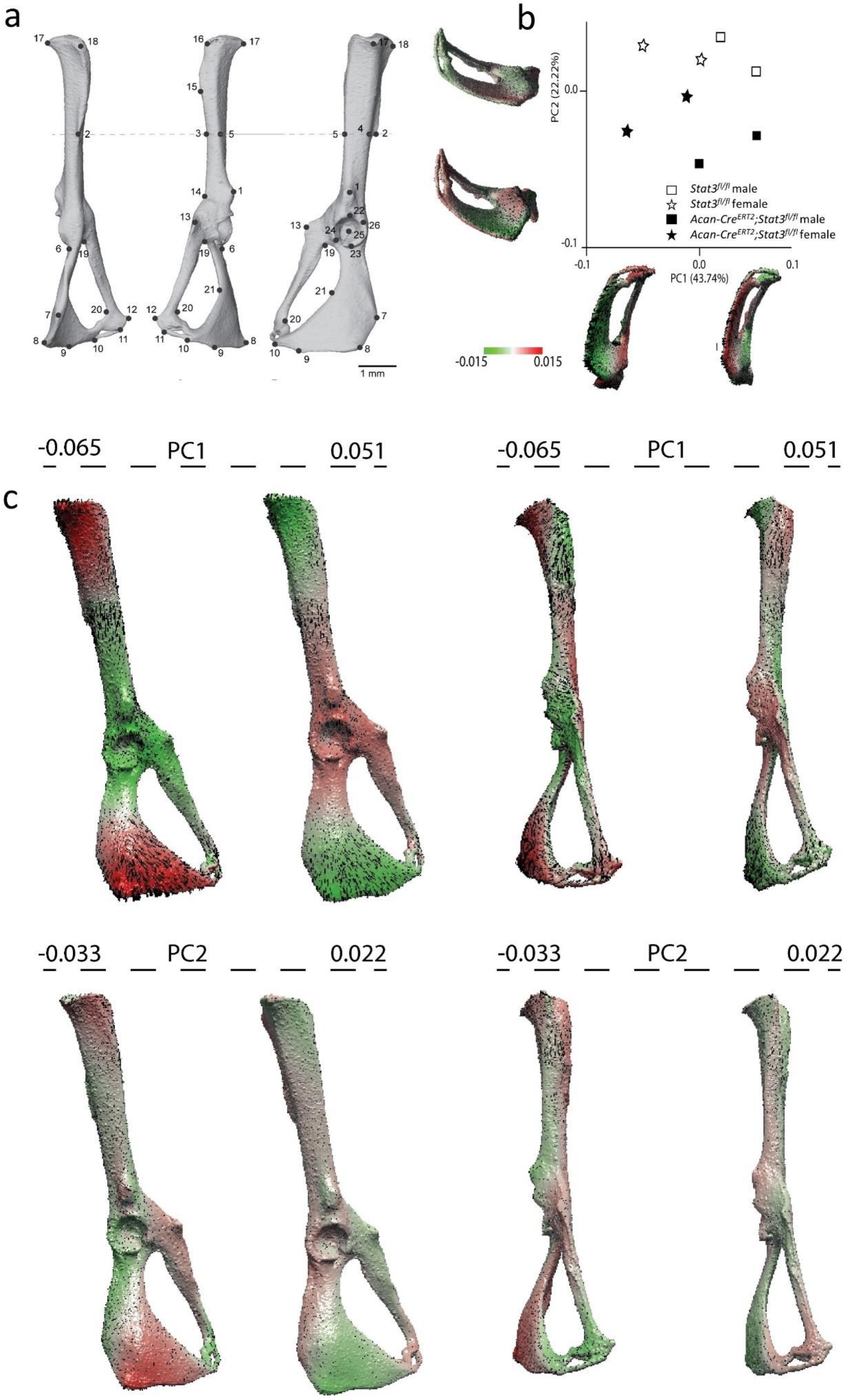
a
b
PC2 (22.22%)
PC1 (43.74%)
$Stat3^{fl/fl}$ male
$Stat3^{fl/fl}$ female
$Acan\text{-}Cre^{ERT2};Stat3^{fl/fl}$ male
$Acan\text{-}Cre^{ERT2};Stat3^{fl/fl}$ female
-0.015
0.015
1 mm
-0.065
PC1
0.051
c
-0.033
PC2
0.022

Supplementary Figure 7: Significant skeletal structural changes result after deletion of *Stat3*. (a) Left pelvis of a male *Acan*-Cre$^{+/ERT2}$;*Stat3*$^{fl/fl}$ mouse in dorsal, ventral and lateral (left to right) views illustrating the 26 landmarks used as a basis for geometric morphometrics. (a) Ventral and dorsal views. (b) Principal component analysis (PCA) of pelvises from Stat3 deleted and control mice based on distances between 26 anatomical landmarks. Animals clearly segregate based on genotype. (c) Deviations along PC1 (above) and PC2 (below) are shown on 3D surface maps of control (left) and Stat3 deleted (right) male pelvic regions. Maps are colored based on degree of deformation with respect to PC values, with red representing features more prominent in control and green more prominent in Stat3 deleted animals, respectively.

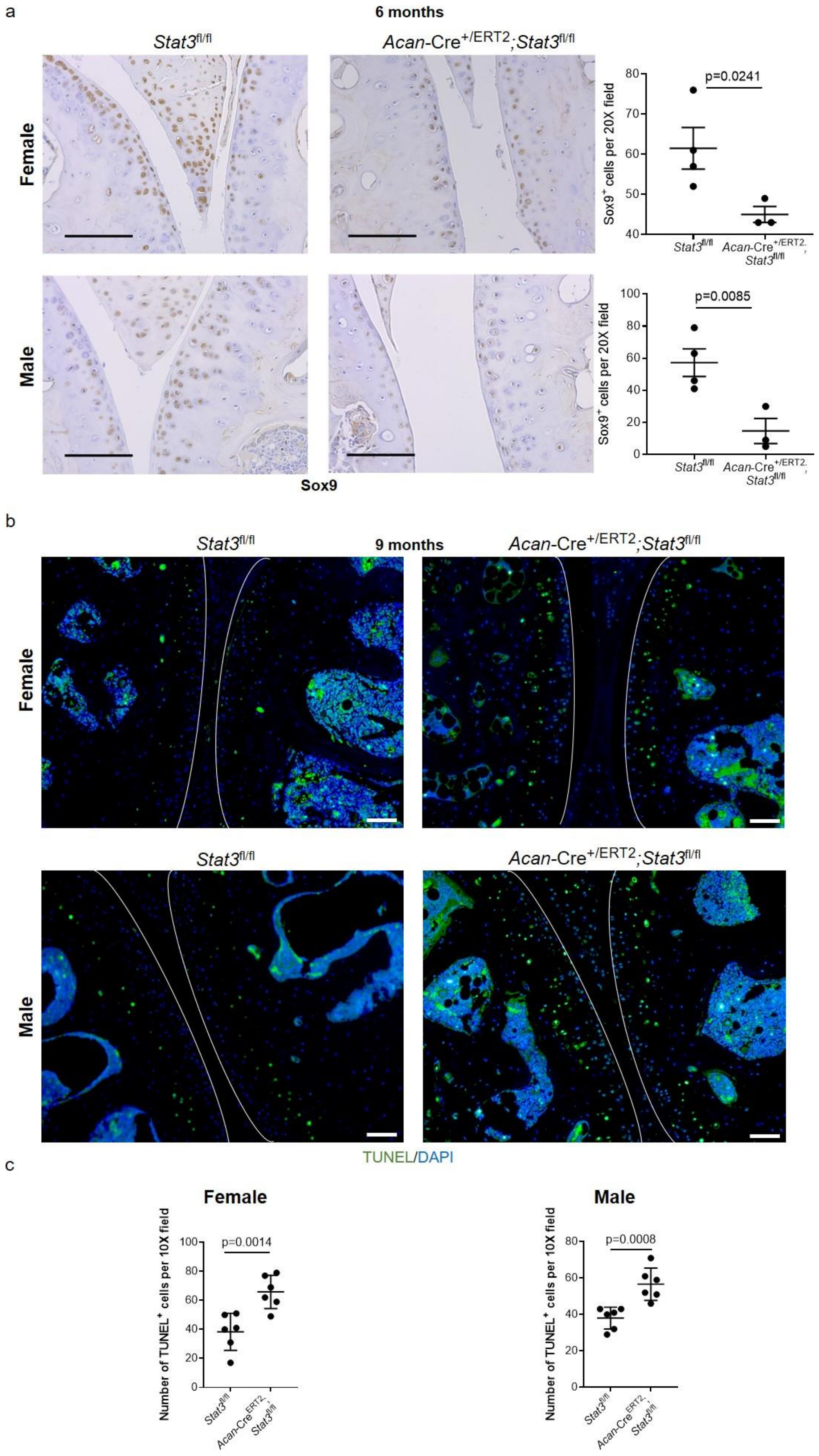
a
6 months
Stat3fl/fl
Acan-Cre+/ERT2;Stat3fl/fl
Female
Male
Sox9+ cells per 20X field
p=0.0241
p=0.0085
Sox9
b
9 months
TUNEL/DAPI
c
Female
Male
Number of TUNEL+ cells per 10X field
p=0.0014
p=0.0008
Stat3fl/fl
Acan-CreERT2;Stat3fl/fl

Supplementary Figure 8: Deletion of *Stat3* results in reduced Sox9 expression and increased apoptosis in articular cartilage. (a) Following tamoxifen administration at P2/3, animals were sacrificed at 6 months. Note reduced frequency and intensity of Sox9 staining in *Acan*-Cre$^{+/ERT2}$;*Stat3*$^{fl/fl}$ animals. Representative images are shown and scale bars = 50 µm; n = 3-4. (b) Assessment of apoptosis in articular cartilage 9 months after *Stat3* deletion by TUNEL staining demonstrated increased cell death (c) in *Acan*-Cre$^{+/ERT2}$;*Stat3*$^{fl/fl}$ animals. The articular surface is delineated by white lines in (b). n = 6; scale bars = 50 µm.

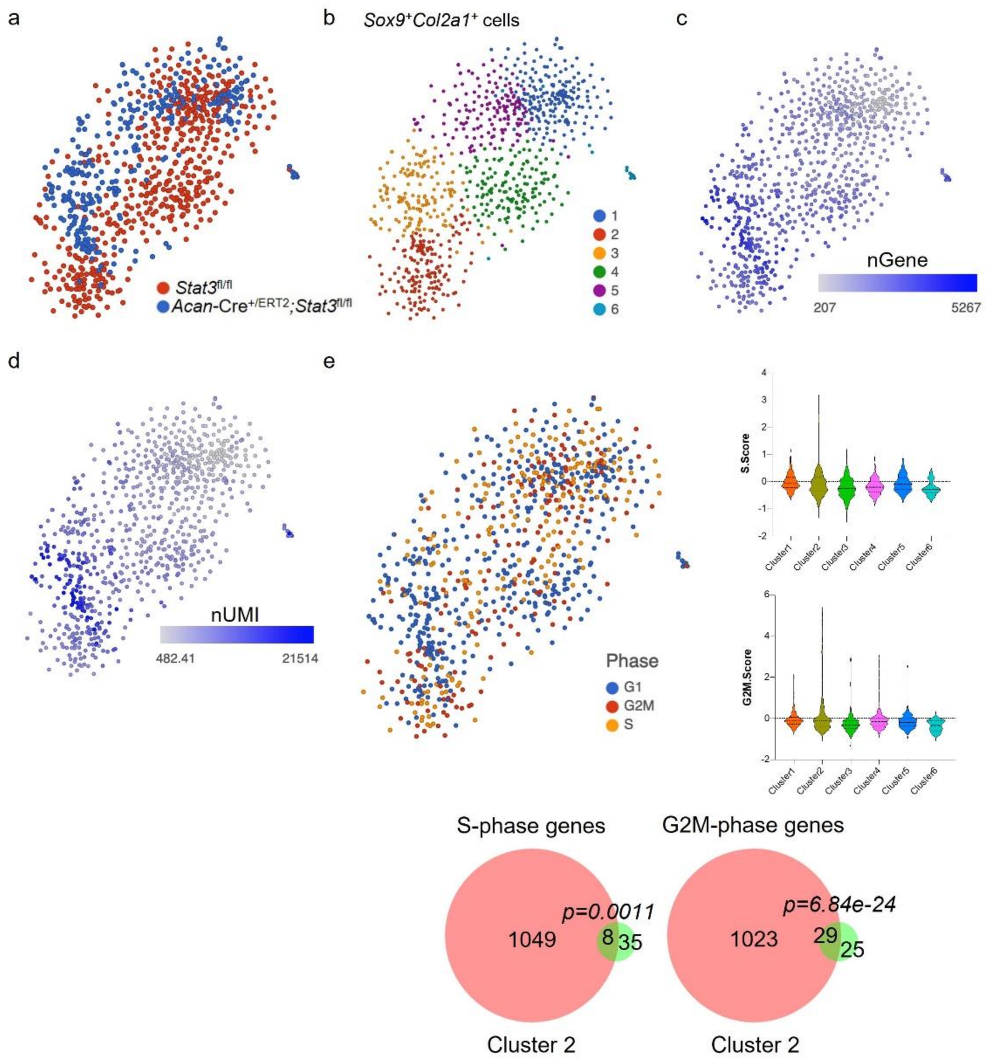

Supplementary Figure 9: Quality control metrics of scRNA-seq confirm clustering is independent of sampling or proliferation status. (a) tSNE plot and (b) k-means clustering of *Sox9*$^{+}$*Col2a1*$^{+}$ cells isolated and pooled from knee joints of 2-3 females of the indicated genotype; see also Figure 6. Quality assessment of the scRNA-seq dataset was conducted by plotting (c) the number of genes (nGene) and (d) the number of unique molecular identifiers (nUMI) detected per cell; cells segregate into clusters interpedently of both metrics. (e) Cell cycle gene set analysis[83] demonstrated significant enrichment of proliferation genes in cluster 2, though all clusters contained cells of varying cell cycle status.

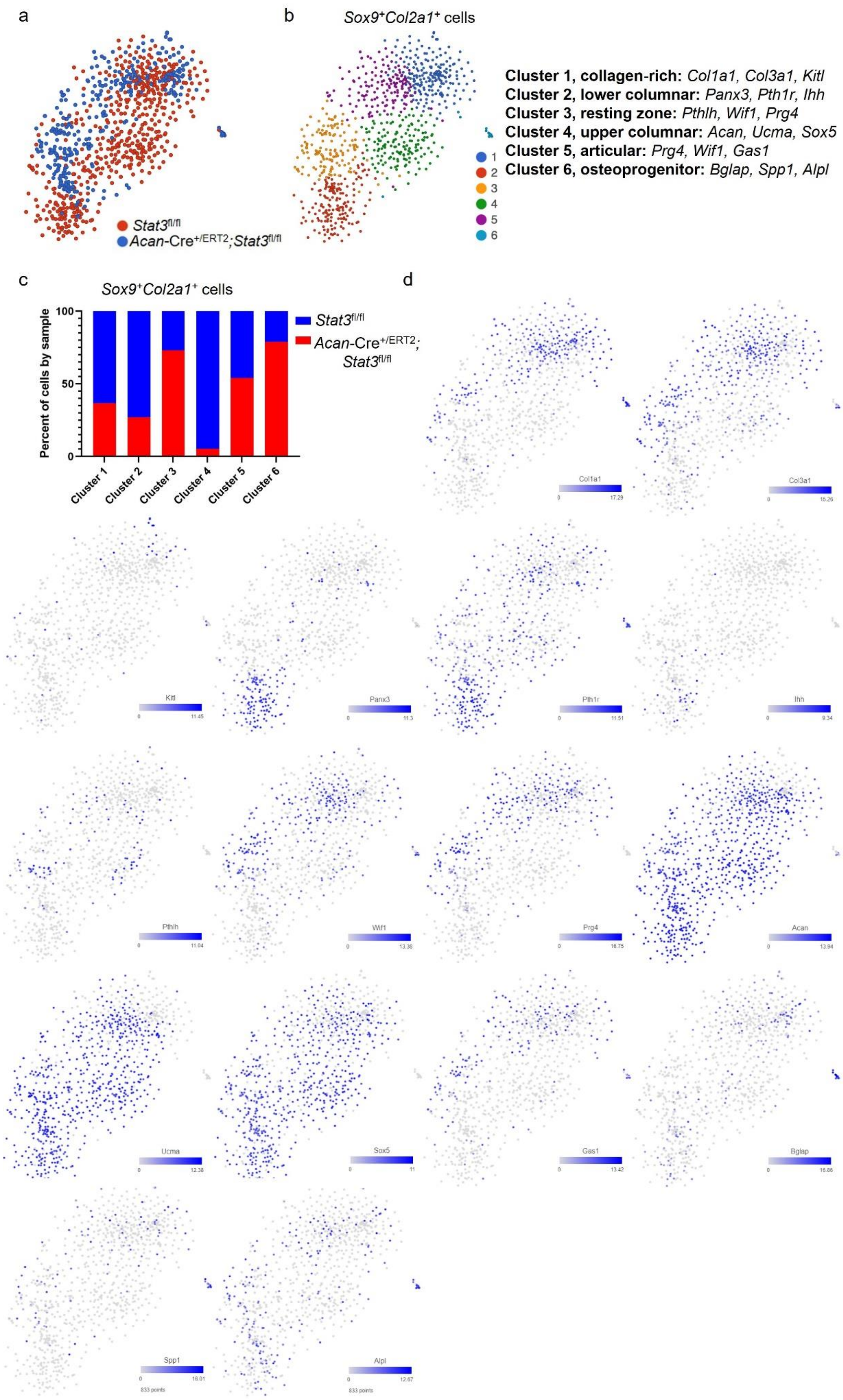
a
b
Sox9+Col2a1+ cells
Stat3fl/fl
Acan-Cre+/ERT2;Stat3fl/fl
Cluster 1, collagen-rich: Col1a1, Col3a1, Kitl
Cluster 2, lower columnar: Panx3, Pth1r, Ihh
Cluster 3, resting zone: Pthlh, Wif1, Prg4
Cluster 4, upper columnar: Acan, Ucma, Sox5
Cluster 5, articular: Prg4, Wif1, Gas1
Cluster 6, osteoprogenitor: Bglap, Spp1, Alpl
1
2
3
4
5
6
c
Sox9+Col2a1+ cells
Percent of cells by sample
100
50
0
Cluster 1
Cluster 2
Cluster 3
Cluster 4
Cluster 5
Cluster 6
Stat3fl/fl
Acan-Cre+/ERT2;
Stat3fl/fl
d
Col1a1
Col3a1
Kitl
Panx3
Pth1r
Ihh
Pthlh
Wif1
Prg4
Acan
Ucma
Sox5
Gas1
Bglap
Spp1
Alpl

Supplementary Figure 10: scRNA-sequencing and unbiased clustering confirm loss of proliferating columnar chondrocytes in the absence of *Stat3*. (a,b) k-means clustering of *Sox9*$^{+}$*Col2a1*$^{+}$ cells yielded 6 clusters prospectively identified based on previously published data[37-39] and the biomarker genes enriched in each cluster. (c) Composition of each cluster based on genotype; clusters 2, 3, 4 and 6 evidenced varying degrees of bias. (d) tSNE plots of biomarkers genes used to speculatively identify each cluster. n=2-3 pooled females of each genotype; see also Figure 6.

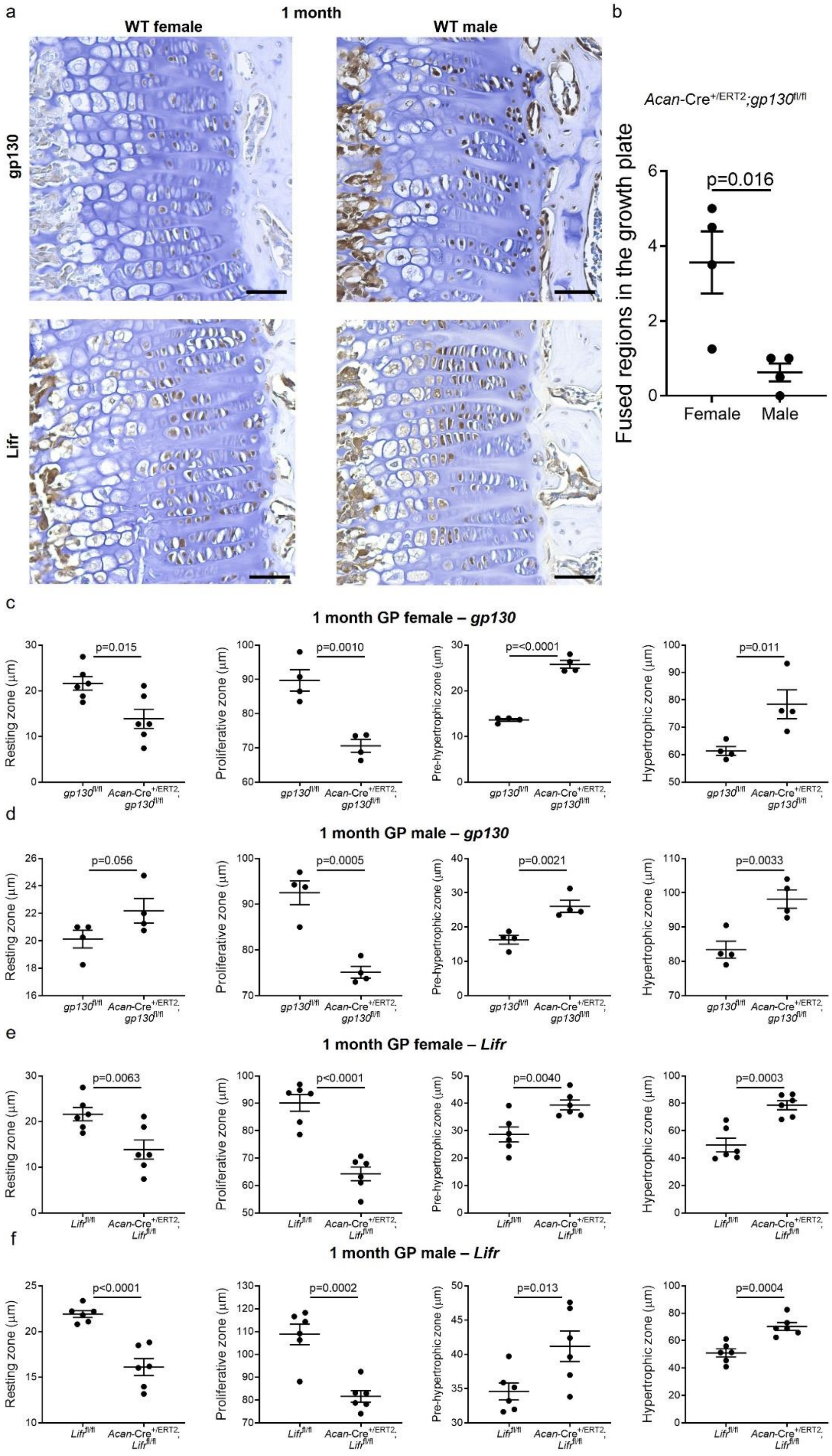

Supplementary Figure 11: Deletion of *gp130* and *Lifr* in chondrocytes resulted in reduced proliferation and increased hypertrophy in the growth plate at 1 month as well as growth

plate fusions. (a) Localization of gp130 and Lifr expression in growth plate chondrocytes in wild type mice. Note the enrichment of both receptors in the proliferative zone. n = 3, representative images are shown; scale bars = 50 µm. (b) *Acan*-Cre$^{+/ERT2}$;*gp130*$^{fl/fl}$ females evidenced significantly more frequent fusions of the growth plate at 3 months versus *Acan*-Cre$^{+/ERT2}$;*gp130*$^{fl/fl}$ males; n = 4. (c-f) Measurement of each zone of the growth plate demonstrated significant reductions in the proliferative zone in both females and males of *Acan*-Cre$^{+/ERT2}$;*gp130*$^{fl/fl}$ and *Acan*-Cre$^{+/ERT2}$;*Lifr*$^{fl/fl}$ animals as compared to controls; in contrast, both the pre-hypertrophic and hypertrophic zones increased in size in both sexes of *gp130* and *Lifr* animals vs. controls. n = 4-6.

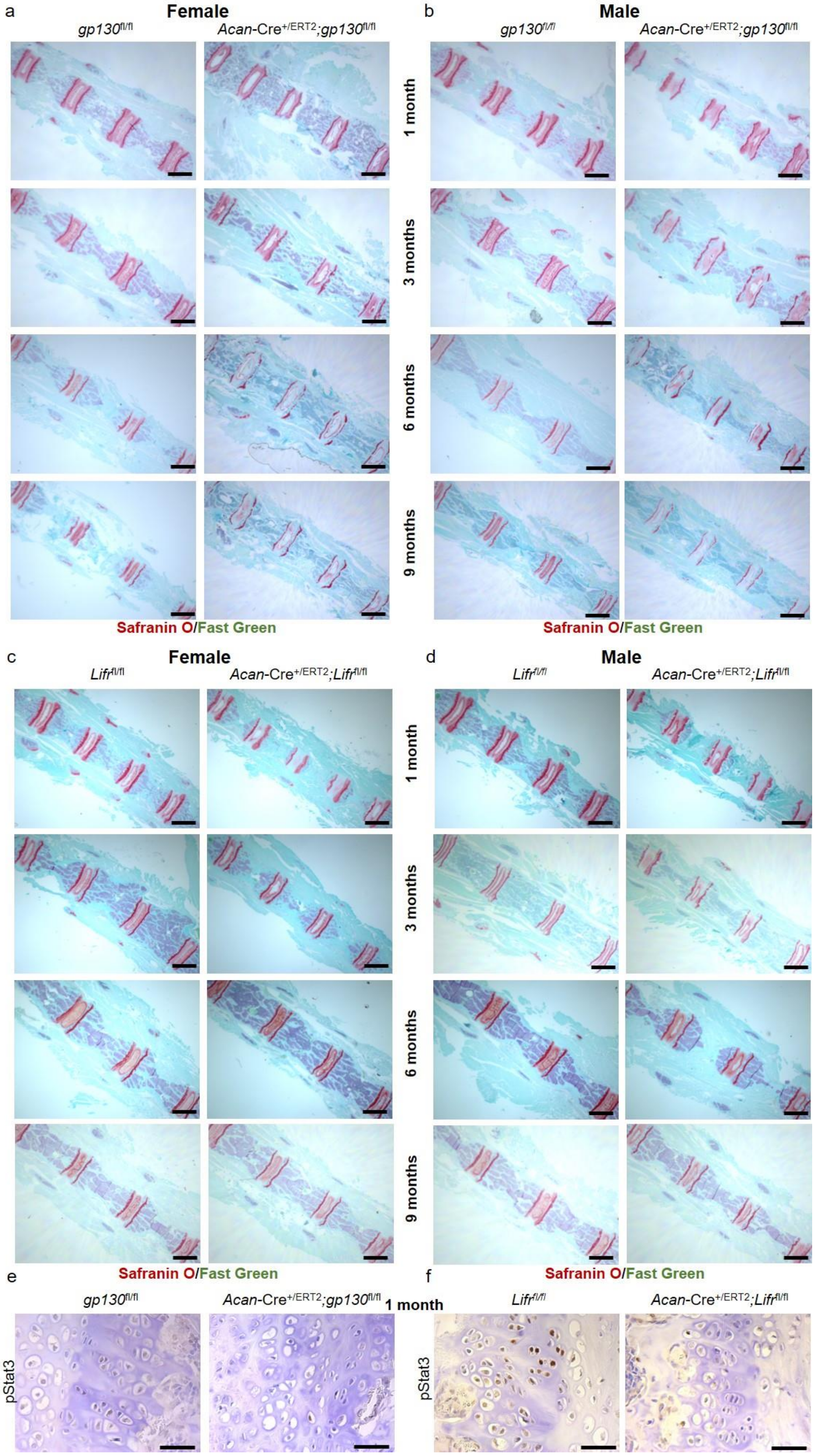
a
Female
$gp130^{fl/fl}$
Acan-Cre$^{+/ERT2}$;$gp130^{fl/fl}$
b
Male
$gp130^{fl/fl}$
Acan-Cre$^{+/ERT2}$;$gp130^{fl/fl}$
1 month
3 months
6 months
9 months
Safranin O/Fast Green
Safranin O/Fast Green
c
Female
$Lifr^{fl/fl}$
Acan-Cre$^{+/ERT2}$;$Lifr^{fl/fl}$
d
Male
$Lifr^{fl/fl}$
Acan-Cre$^{+/ERT2}$;$Lifr^{fl/fl}$
1 month
3 months
6 months
9 months
Safranin O/Fast Green
Safranin O/Fast Green
e
$gp130^{fl/fl}$
Acan-Cre$^{+/ERT2}$;$gp130^{fl/fl}$
1 month
f
$Lifr^{fl/fl}$
Acan-Cre$^{+/ERT2}$;$Lifr^{fl/fl}$
pStat3
pStat3

Supplementary Figure 12: Postnatal deletion of *gp130*, and to a lesser extent *Lifr*, affects vertebral growth plates and bodies. (a) Coronal sections of caudal vertebrae of *Acan*-Cre^{ERT2};*gp130*$^{fl/fl}$ and control *gp130*$^{fl/fl}$ mice demonstrate shorter vertebral bodies in both female and (b) male mice. Note the increased number of vertebrae visible in each image in the *gp130* deleted mice vs. controls, as well as the degeneration of the growth plates. Deletion of *Lifr* resulted in a milder phenotype, with moderate compression of the caudal vertebrae in both females (c) and males (d); degeneration of the growth plates is not obvious until the 9 month timepoint. In a-d, scale bars = 1 mm. (e) Deletion of both *gp130* and *Lifr* (f) resulted in substantial loss of Stat3 activity (pStat3) in vertebral growth plates at 1 month; scale bars = 50 µm. For all panels, n = 4. Representative images are shown.

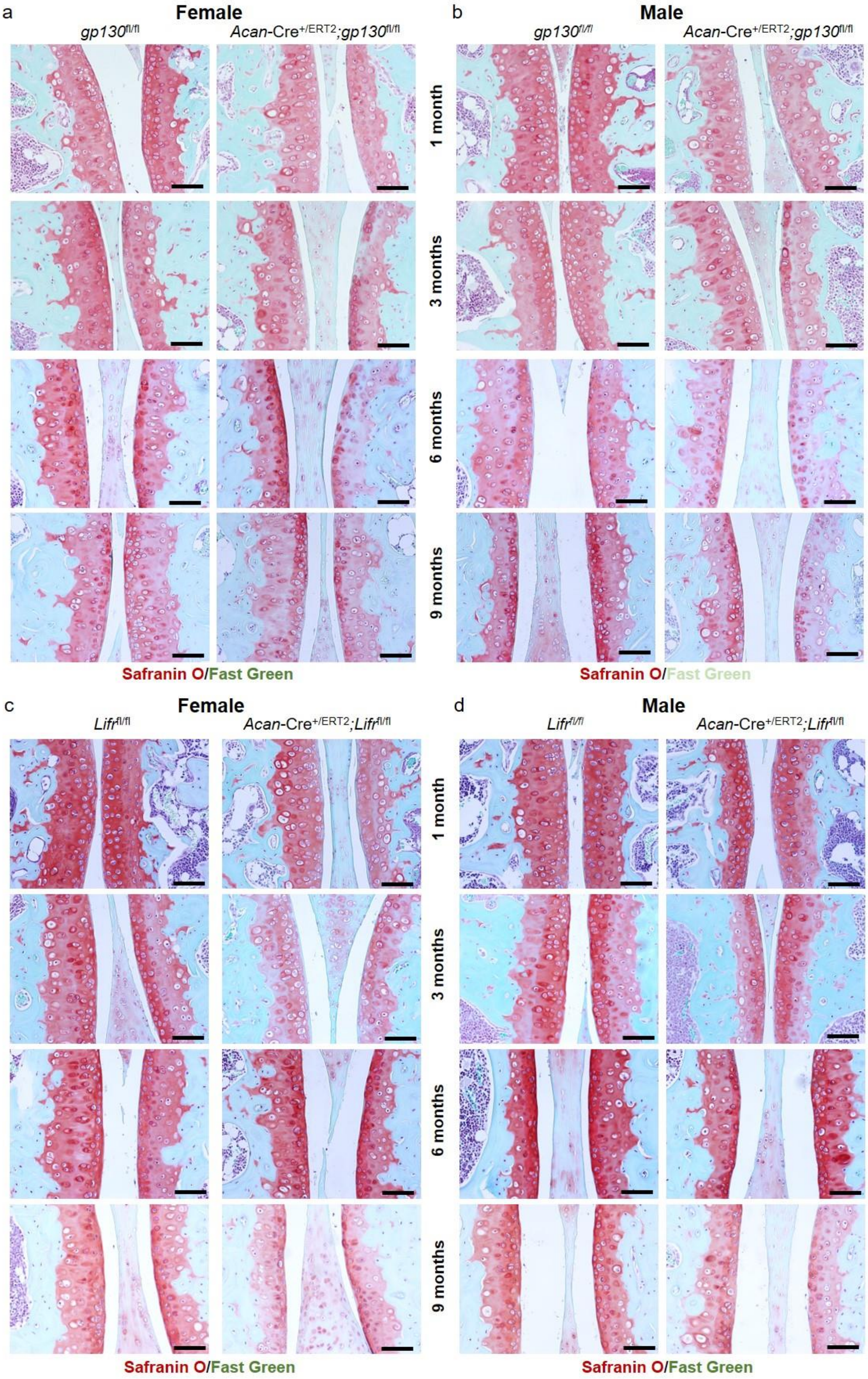
a
Female
gp130fl/fl
Acan-Cre+/ERT2;gp130fl/fl
b
Male
gp130fl/fl
Acan-Cre+/ERT2;gp130fl/fl
1 month
3 months
6 months
9 months
Safranin O/Fast Green
Safranin O/Fast Green
c
Female
Lifrfl/fl
Acan-Cre+/ERT2;Lifrfl/fl
d
Male
Lifrfl/fl
Acan-Cre+/ERT2;Lifrfl/fl
1 month
3 months
6 months
9 months
Safranin O/Fast Green
Safranin O/Fast Green

Supplementary Figure 13: Postnatal *gp130*, and to a lesser extent *Lifr*, deletion results in subtle changes in articular cartilage of the knee joint. (a) Administration of tamoxifen at P2/P3 to *Acan*-Cre^{ERT2};*gp130*$^{fl/fl}$ and control *gp130*$^{fl/fl}$ female and (b) male mice elicited reduced proteoglycan staining in articular cartilage at 6 and 9 months of age in both sexes. Deletion of *Lifr* resulted in a milder and delayed phenotype, with proteoglycan depletion not evident until 9 months of age in both females (c) and males (d). For all panels, representative images are shown; n = 4 and scale bars = 50 µm.

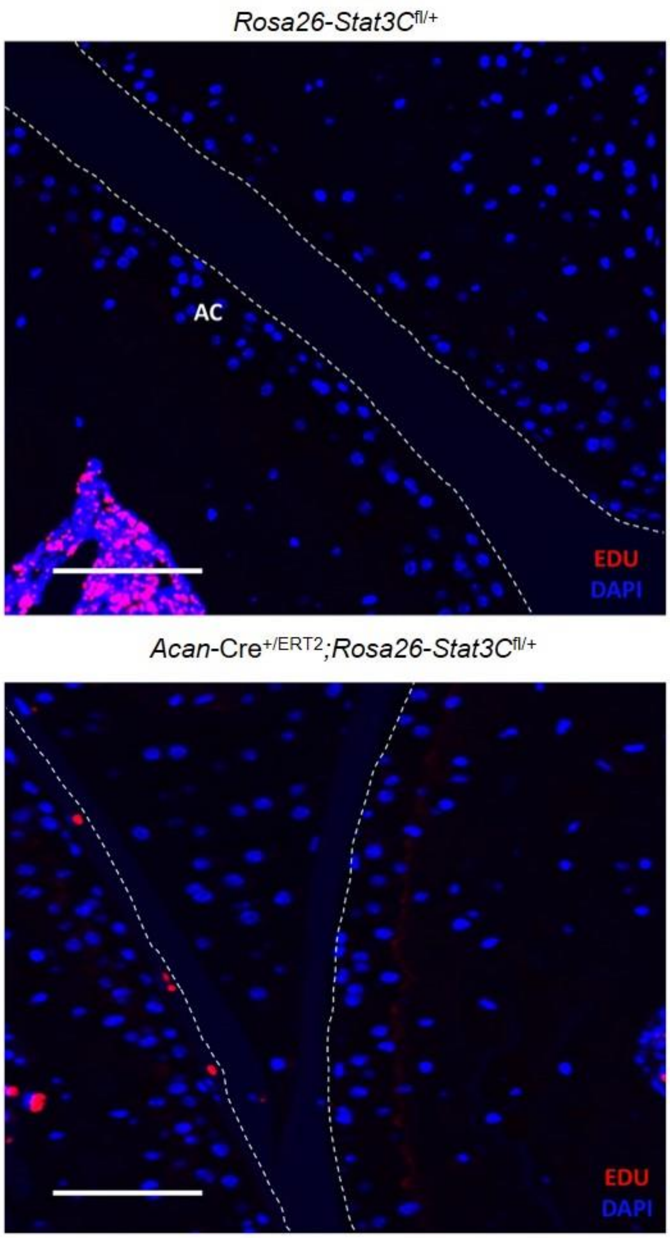

Supplementary Figure 14: Increased proliferation in articular cartilage following overexpression of constitutively active Stat3. EdU was injected intraperitoneally each day for 4 days before harvesting knee joints at 1 month of age. Representative images of the articular cartilage (AC) are shown. Scale bars = 50 μm; n=5 for each group.

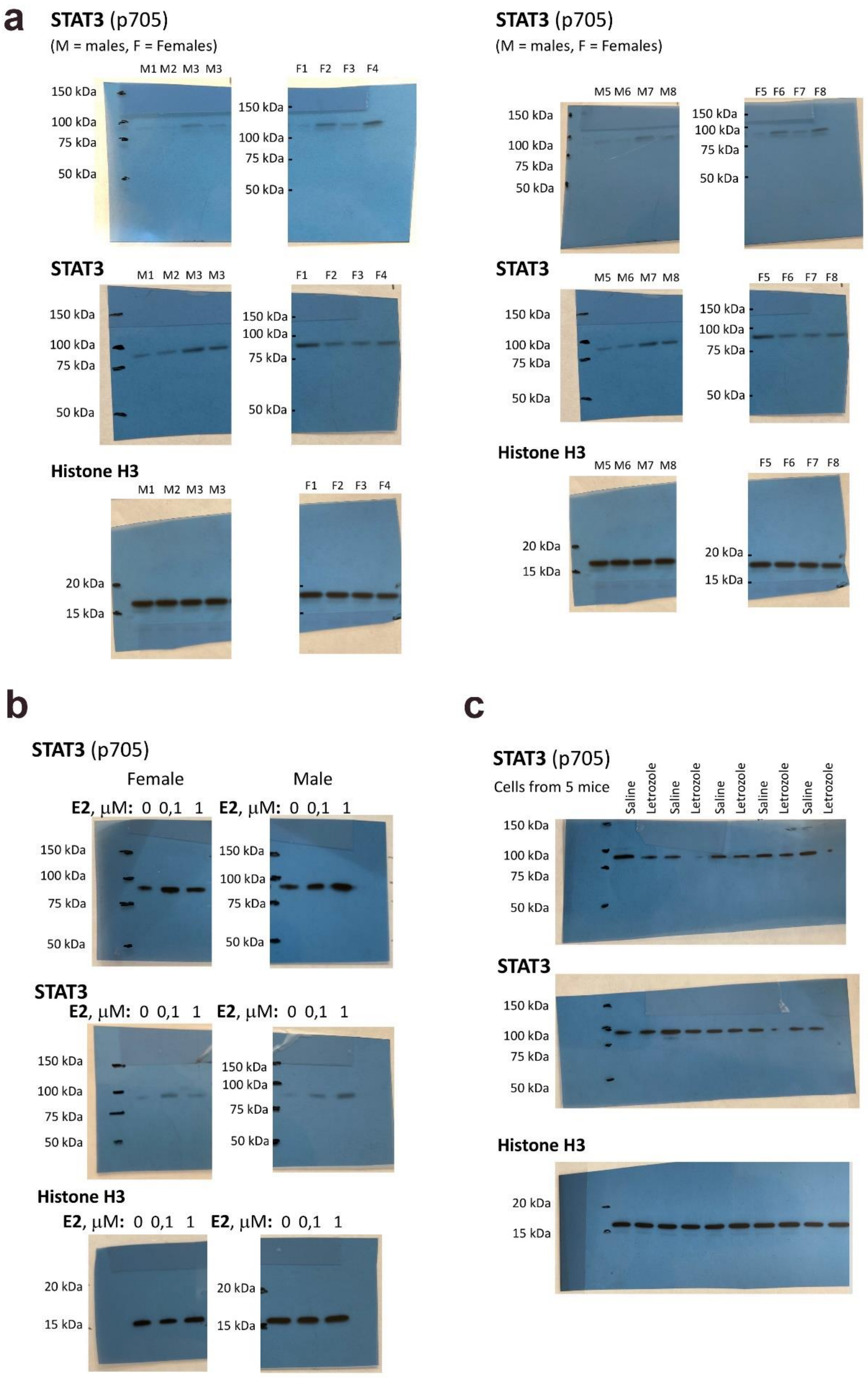
a
STAT3 (p705)
(M = males, F = Females)
M1 M2 M3 M3
F1 F2 F3 F4
150 kDa
100 kDa
75 kDa
50 kDa
STAT3
Histone H3
20 kDa
15 kDa
M5 M6 M7 M8
F5 F6 F7 F8
b
STAT3 (p705)
Female
Male
E2, µM: 0 0,1 1
STAT3
Histone H3
c
STAT3 (p705)
Cells from 5 mice
Saline
Letrozole
Saline
Letrozole
Saline
Letrozole
Saline
Letrozole
Saline
Letrozole
STAT3
Histone H3

Supplementary Figure 15: Images of uncut Western blots. (a) Data for Figure 1f. (b) Data for Figures 10a and 10b. (c) Data for Figure 10c.
