# Supplementary material for: gp130/STAT3 signaling is required for homeostatic proliferation and anabolism in postnatal growth plate and articular chondrocytes
Source: Commun Biol. 2022 Jan 17;5:64. doi: 10.1038/s42003-021-02944-y (PMC8763901; doi:10.1038/s42003-021-02944-y)
Supplement: Supplementary file 2 — Description of Additional Supplementary Files [file 42003_2021_2944_MOESM2_ESM.pdf]

Description of Additional Supplementary Files

File name: Supplementary Data 1
Description: Knockdown of STAT3 in human fetal chondrocytes revealed significant changes in expression of genes encoding matrix proteins. Negative fold changes indicate enrichment in control shRNA cells, while positive fold changes nominate genes enriched in STAT3 shRNA cells.

File name: Supplementary Data 2
Description: Average values of lumbar (L) vertebral body height and endplate areas as determined by microCT. Average value ± standard deviation is shown; n=4 males and females of each genotype pooled. Data were generated based on the measurements described in Supplementary Table 3. VVBH = Ventral (anterior) vertebral body height, DVBH = Dorsal (posterior) vertebral body height, CSA = Cranial endplate surface area, CaSA = Caudal endplate surface area. Bold = $p < 0.001$, italics = $p < 0.01$, underline = $p < 0.05$ and normal text = not significant.

File name: Supplementary Data 3
Description: Source data files for all main figures.
